# Assessment of magnitude and associated factors of attitude towards time management among health professionals working in public hospitals of Dessie City, Northeast Ethiopia

**DOI:** 10.1186/s12913-023-10005-9

**Published:** 2023-09-09

**Authors:** Ali Yimer, Mohammed Ahmed, Amare Zewdie, Sefineh Fenta

**Affiliations:** 1 Department of Public Health, College of Health Sciences, Woldia University, Woldia, Ethiopia; 2 Department of Public Health, College of Medicine and Health Sciences, Wolkite University, Wolkitie, Ethiopia

**Keywords:** Attitude towards time management, Health professionals, Public hospitals, Dessie City, Ethiopia

## Abstract

**Background:**

Good attitude towards to time management is the backbone to bring a change at individual and organizational levels in different sectors across the globe. But it has been ignored by different institutions, particularly in low and middle-income countries including Ethiopia. However, this can be alleviated if there are punctual, committed, and accessible health professionals that could translate the national aspirations and the desire of the community into reality. This study aims to determine the magnitude and associated factors of attitude towards time management among health professionals working in public hospitals of Dessie City, Northeast Ethiopia,

**Methods:**

Institution-based cross-sectional study was conducted from March 24 –April 24, 2021 among 409 health professionals using a stratified sampling technique, Frequency and percentage were used to describe the study population. Multivariable logistic regression analysis was used to identify independent predictors. A p-value of < 0.05 with 95% CI were used to declare statistically significant associations.

**Results:**

The overall good time management attitude among sample was 67%(95%CI:66.77–67.22%). Satisfaction with organizational policy and strategy (AOR: 2.69, 95%CI: 1.42–5.09), satisfaction with supervisor support (AOR: 2.12, 95% CI: 1.19–3.77), and managers’ good attitude towards time management (AOR: 2.00, 95%CI: 1.23–3.25) were significantly associated with good attitude towards time management among health professionals.

**Conclusion:**

The attitude towards time management in public hospitals of Dessie City was low. Satisfaction with organizational policies and strategies, satisfaction with supervisor support, and managers’ good attitude towards time management were delineated factors. This low attitude towards time management could affect the practice and it compromise the health service coverage and quality unless timely and appropriate interventions should be taken. Strengthening strategies aimed at maximizing job satisfaction and emphasizing an attitude towards time might have a substantial contribution.

## Background

Effectiveness and profitability in one task can be achieved through time, which is a special resource that can’t store or save for later use [1–3]. Time management is the management of life, and also can be seen as “self-management”, the skill of establishing smart decisions regarding how to spend our time to accomplish set goals and improving our productivity [4] [5]. Time management emphasizes the principle that it is more relevant to perform the right things than to do things right to accomplish goals [6, 7].

A fraction of microseconds matters for health professionals as far as they deal with a variety of health problems to be responsive to the health needs of the patient and society at large [8–10]. Time management skills enable health professionals to get professional satisfaction and reduce stress, enhance career satisfaction, greater work–life balance, and long-term growth [9]. The implementation of sound management practices is a key point for maximizing productivity and quality. However, a good attitude towards time management (ATM) is the base for effective time management practice (TMP) [11]. It is useful to know what portion of time is spent on certain activities by health professionals to give the right service to the right patient at the right time [1].

The overall goals of the World Health Organization [12] health system building blocks of health care are health improvement, responsiveness, and social and financial risk protection [12]. Moreover, the vision of the newly revised 2017 Health Policy of the Ethiopian Government is to see a healthy, productive, and prosperous generation [13]. The stated goals and vision will only be possible by punctual, committed, and accessible health professionals that could translate the national aspirations and the desire of the community into a reality [12].

The developed nations have a strong ATM and invested much more time on their work, which leads to rapid economic growth across the globe [14]. However, what is sad is the existence of the poor culture of managing time in many institutions, especially in low and middle-income countries including Africa [15, 16]. The lack of good ATM could lead to ineffective time management practice which in turn lead to uncontrollable stress, poor work quality, inefficient workflow, poor professional reputation, and a stalled career [17, 18].

Although time is the key to the development of the health system and the development of the nation, little emphasis has been given by the concerned bodies. Little has been done at health facilities, regional and national levels to maximize effective time management in Ethiopia. The absence of good ATM in every organization, particularly health system organizations, is still an existing problem that is the bottleneck for the success of employees [14].

Some studies have been done on TMP [19–21]. However, ATM among health professionals in health sectors is not addressed yet. Therefore; this study aims to assess the magnitude and associated factors of attitude towards time management among health professionals working in Public hospitals of Dessie City, northeast Ethiopia.

## Materials and methods

### Study Design and setting

A cross-sectional study was conducted from March 24 to April 24/2021 at public hospitals in Dessie city. Dessie city is one of the metropolitan cities in Amhara National Regional State, which lies at an altitude of 2470 m above sea level. It is surrounded by the imposing Tossa Mountain that overlooks the city in the west. It is located 401 km far from Addis Ababa, the capital city of Ethiopia, and 488 km from Bahir Dar, the capital city of Amhara National Regional State. It has two Public Hospitals, eight public Health Centers, and five General Private Hospitals serving Dessie City and the surrounding more than 8 million people of the catchment area (East Amhara, part of Tigray and Afar Region). There are 675 health professionals in Dessie Public Hospital.

### Source and study Population

All health professionals working in Dessie City public hospitals were source population, whereas randomly selected health professionals who fulfilled the inclusion criteria in Dessie City public hospitals. All full-time employee health professionals who are available during the study period and reside at least for 6 months were included.

### Sample size estimation and sampling technique

The sample size was determined by using a single population proportion formula by considering an assumption of the magnitude of 50%, hence there is no previous study in public Hospitals, 5% margin of error, 95% confidence level, and 10% nonresponse rate. The sample size was calculated using the single population proportion formula as follows.

$${\rm{n}}\,{\rm{ = }}\,{\left( {{{\rm{z}}_{{\rm{\alpha /2}}}}} \right)^{\rm{2}}}{\rm{p}}\left( {{\rm{1 - p}}} \right){\rm{/}}{{\rm{d}}^{\rm{2}}}{\mkern 1mu} {\mkern 1mu} {\mkern 1mu} {\mkern 1mu} {\mkern 1mu} {\mkern 1mu} {\mkern 1mu} {\mkern 1mu} {\mkern 1mu} {\mkern 1mu} {\mkern 1mu} {\mkern 1mu} {\mkern 1mu} {\mkern 1mu} {\mkern 1mu} {\mkern 1mu} {\mkern 1mu} {\mkern 1mu} {\mkern 1mu} {\mkern 1mu} {\mkern 1mu} {\rm{n}}\,{\rm{ = }}\,{\left( {{\rm{1}}{\rm{.96}}} \right)^{\rm{2}}}{\rm{(0}}{\rm{.5)}}\left( {{\rm{0}}{\rm{.5}}} \right){\rm{/0}}{\rm{.0}}{{\rm{5}}^{\rm{2}}}$$

$${{\rm{Z}}_{{\rm{\alpha /2}}}}{\rm{ = }}\,{\rm{confidence}}{\mkern 1mu} \,{\rm{interval}}{\mkern 1mu} \,{\rm{at}}{\mkern 1mu} \,{\rm{95\% ,}}{\mkern 1mu} \,\,\,\,\,{\rm{n}}\,{\rm{ = }}\,{\rm{384}}$$

$${\rm{d}}\,{\rm{ = }}\,{\rm{level}}\,{\rm{of}}\,{\rm{significance}}\,{\rm{at}}\,{\rm{5\% }}$$

$${\rm{p}}\,{\rm{ = }}\,{\rm{population}}\,\,{\rm{proportion}}\,{\rm{50\% }}$$

After adding a 10% non-response rate, the total sample size was 422.

The samples were selected from seven departments by stratified sampling technique using profession as strata. To select 422 health professionals from Dessie City public hospitals, each hospital (Dessie Specialized Referral Hospital and Boru Meda General Hospital) health professionals’ payroll from the Human Resource Management was used as a frame for each professional category, and then the number of hospital employees in each profession was proportionally allocated to the sample size. Finally; the study subjects of each profession were selected by using a simple random sampling technique from a total of 422 health professionals in public hospitals.

### Study variables and measurements

The dependent variable was **ATM (Good/Poor)**, which was defined as the likelihood of employees’ intention on effective time management utilization. It was measured by three items having a Likert scale out of five points. If the participant’s response score was above the mean, it was represented as a good attitude otherwise poor attitude towards ATM [3].

#### Socio-demographic factors

age, sex, marital status, educational status, work experience, type of profession, monthly salary, having less than 18 years old children, and work unit.

#### Job satisfaction factors

physical work environment, co-worker relationship, `workload, performance appraisal, organizational policy and strategy, compensation and benefit, work autonomy, supervisor support, professional development, recognition and reward, and managers’ attitude towards time were independent variables of the study.

**Managers’ attitude towards time**: indicates the managers’ outlook regarding time management. It was measured using five items having a five-point Likert scale. If the participant’s response scored above the mean value, it was represented as a good attitude if not a poor attitude [3].**Job satisfaction**: the extent of positive or negative views workers hold about their jobs. It was measured using thirty-eight items, each having a five-point Likert scale ranging from 1 strongly dissatisfied to five strongly satisfied using Minnesota satisfaction measurements [22].

#### Dimensions of satisfaction

the sum scores of each item having values above the mean of the sum scores were regarded as satisfied and if it was below or equal to the mean of the sum scores classified as unsatisfied [23–25].

### Data collection instruments and procedures

Data were collected using a structured, pre-tested, and self-administered questionnaire which was adapted from different literatures [19–21, 26, 27]. The data were collected by five trained diploma nurses and also supervised by three nurses.

### Data Quality Control

The questionnaire was pretested by taking 5% of the sample size at Woldia General Hospital. Amendments regarding the instrument such as unclear questions and ambiguous words were checked. Data collectors and supervisors were recruited based on their experience in research and one-day training was given on the objective of the study instrument and data collection procedures by the principal investigator. Furthermore, the tools were reviewed by public health research experts. Supervision was conducted by the principal investigator and supervisors. To ensure data quality, each data collector checked the questionnaire from each study participant for completeness daily. The supervisors and principal investigator reviewed each questionnaire daily and checked for completeness.

### Data processing and analysis

Data were checked for completeness and consistency, then compiled and coded. Finally, it was entered into Epidata version 4.6, and exported to SPSS version 25 statistical software. Frequencies and cross-tabulations were used to summarize descriptive statistics of the data and tables were used for data presentation. Significance of association was determined using crude and adjusted odds ratio with 95% confidence interval. Bivariate analysis using logistic regression model was used to select candidates (variables with a p-value < 0.2) for multivariable logit model. After multivariable logit, variables which have a p-value of < 0.05 with a 95% confidence interval were regarded as independent factors significantly associated with ATM u. The internal consistency of the tool was checked and the scored a Cron-Batch Alpha value of above 0.7.

## Results

### Socio-demographic profile

A total of 409 participants responded to the self-administered questionnaire with a 96.9% response rate. About, 235(57.5%) of the study participants were male. The median ± IQR age of the study participants was 29 years ± 4 years. About, 61.6% of the respondents were married. About, 71.6% of participants lived with their families. The median monthly salary in the sample was 7071 ± 1985 ETB **(**Table 1**)**. Moreover,58.9% of participants had reported good managers’ attitudes toward time management in public hospitals.

**Table 1:** Socio-demographic characteristics of health professionals working in public hospitals of Dessie City, Ethiopia, 2021

Variable	Category	Frequency (%)
Sex	Male	235(57.5)
	Female	174(42.5)
Age	20–24 years	58(14.2)
	25–29 years	179(43.8)
	30–34 years	96(23.5)
	Above 35 years	76(18.5)
Marital status	Unmarried	157(38.4)
	Married	252(61.6)
Education level	Diploma	67(16.4)
	Degree and above	342(83.6)
Work experience	0–5 years	250(61.1)
	6–10 years	119(29.1)
	Above 10 years	40(9.8)
Living condition	With family	293(71.6)
	Without family	116(28.4)
Profession	Nurse	267(65.3)
	Doctor	57(13.9)
	Laboratory professionals	37(9)
	Pharmacy	35(8.6)
	Anesthesia and Radiology	13(3.2)
Monthly income	below 6193 ETB	124(30.3)
	6193–7071 ETB	165(40.3)
	> 7071 ETB	120(29.4)
Work unit	Outpatient	202(49.4)
	Inpatient	207(50.6)
Having < 18 years old child	Yes	183(44.7)
	No	226(55.3)

n = sample size,%=percentage

### Job satisfaction factors

Three hundred sixty- three(85.8%) of health professionals were satisfied with co-worker relationships in public hospitals. On the other hand,86.1%,80%,80.2%, and 63.1% of health professionals were dissatisfied with compensation and benefits, professional development, recognition and reward, and workload, respectively. **(**Table 2**)**.

**Table 2:** Level of job satisfaction by different dimensions among health professionals working in public hospitals of Dessie City, Ethiopia, 2021

Variable	Category	Frequency (%)
Organizational policies and strategies	Unsatisfied	247(60.4)
	Satisfied	162(39.6)
Performance appraisal	Unsatisfied	237(57.9)
	Satisfied	172(42.1)
Compensation and benefit	Unsatisfied	352(86.1)
	Satisfied	57(13.9)
Physical working environment	Unsatisfied	295(72.1)
	Satisfied	114(27.9)
Professional development	Unsatisfied	327(80.0)
	Satisfied	82(20.0)
Supervisor support	Unsatisfied	208(50.9)
	Satisfied	201(49.1)
Work autonomy	Unsatisfied	186(45.5)
	Satisfied	223(54.5)
Work load	Low	258(63.1)
	High	151(36.9)
Recognition and reward	Unsatisfied	328(80.2)
	Satisfied	81(19.8)
Co-worker relationship	Unsatisfied	58(14.2)
	Satisfied	351(85.8)

n = sample size,%=percentage

### The magnitude of ATM

The overall good ATM in public hospitals was 67% (95% CI: 66.77–67.22%).

### Factors associated with ATM in public hospitals of Dessie City

In the bivariate logistic regression analysis, twenty variables were included and fourteen of them had a p-value of less than 0.2. In multivariable logit model, three variables were remained statistically significant. The finding indicated that, health professionals who were satisfied with organizational policies and strategies were 2.69 times more odds to have good ATM than unsatisfied respondents (AOR: 2.69, 95%CI: 1.42–5.09). Moreover, professional who were satisfied with supervisor support were 2.12 times more odds to have good ATM compared with their counterparts (AOR: 2.12, 95%CI: 1.19–3.77). Finally, health professionals who had good managers’ attitudes towards time management were 2.00 times more odds to have good ATM than their counterparts (AOR: 2.00, 95%CI: 1.23–3.25) **(**Table 3**)**.

**Table 3:** Bivariate and multivariate logistic regression analysis of factors associated with ATM among health professionals working in public hospitals of Dessie City, Ethiopia, 2021

Variables	Category	ATM		COR (95% CI)	AOR (95% CI)
		Good	Poor		
Sex	Male	168	67	1.61(1.06–2.44) *	1.56(0.95–2.54)
	Female	106	68	1	1
Age	20–24 years	37	21	1	1
	25–29 years	118	61	1.10(0.59–2.04)	1.05(0.51–2.19)
	30–34 years	66	30	1.25(0.63–2.48)	1.00(0.42–2.38)
	above 35 years	53	23	1.31(0.63–2.70)	0.95((0.33–2.73)
Education	Diploma	41	26	1	1
	Degree and above	233	109	1.36(0.79–2.33)	1.16(0.61–2.19)
Work experience	0–5 years	163	87	1	1
	6–10 years	87	32	1.45(0.90–2.35)	1.74(0.98–3.09)
	above 10 years	24	16	0.80(0.40–1.59)	0.92(0.33–2.57))
Profession	Nurse	178	89	1	1
	Doctor	45	12	1.88(0.94–3.72)	1.70(0.77–3.73)
	Laboratory professionals	25	12	1.04(0.50–2.17)	1.26(0.52–3.04)
	Pharmacy	21	14	0.75(0.36–1.55)	0.82(0.34–1.94)
	Anesthesia and Radiology	5	8	0.31(0.10–0.98)*	0.64(0.18–2.33)
Work unit	Outpatient unit	150	52	1	1
	Inpatient unit	124	83	0.52(0.34–0.79)	0.60(0.36–1.02)
Organizational policies and strategies	Unsatisfied	141	106	1	1
	Satisfied	133	29	3.45(2.15–5.54)**	2.69(1.42–5.09)*
Performance appraisal	Unsatisfied	148	89	1	1
	Satisfied	126	46	1.65(1.07–2.53)**	0.81(0.46–1.43)
Compensation and benefit	Unsatisfied	227	125	1	1
	Satisfied	47	10	2.59(1.26–5.30)	0.98(0.39–2.48)
Professional development	Unsatisfied	210	117	1	1
	Satisfied	64	18	1.98(1.12–3.50)*	0.87(0.42–1.80)
Supervisor support	Unsatisfied	113	95	1	1
	Satisfied	161	40	3.38(2.18–5.26)*	2.12(1.19–3.77)*
Work autonomy	Unsatisfied	110	76	1	1
	Satisfied	164	59	1.92(1.27–2.92)**	1.10(0.65–1.86)
Recognition and reward	Unsatisfied	209	119	1	1
	Satisfied	65	16	2.31(1.28–4.18)	1.23(0.56–2.71)
managers’ attitudes to TM	poor attitude	95	73	1	1
	good attitude	179	62	2.22(1.46–3.38)*	2.00(1.23–3.25)*

COR: crude odds ratio; CI: confidence interval; AOR: adjusted odds ratio; 1: reference category;*: significant at p < 0.05;**: significant at p < 0.001; n: sample size

## Discussion

The present study revealed that 67% (95%CI:66.77–67.22%) of professional had a good ATM in public hospitals of Dessie City. But, health professionals are expected to have nearly about 100% ATM.

According to this study, health professionals’ ATM could be impaired by job satisfaction dimensions regarding organizational policy and strategy. This finding was supported by the job satisfaction theory of Adam’s equity which stated that “if an employee perceives that he/she is not fairly treated with organizational policies and strategies, he/she will not be happy and so slow the pace of performance” [28] This similarity could be justified due to the reason that health professionals who were satisfied with the policy and strategy of their organization might have a strong attachment to their institution [29]. This feeling may enable them to develop trust and ownership; in turn, which makes them punctual for their job.

Similarly, satisfaction with supervisor support also an input to have good ATM as per the this study This finding was in agreement with Herzberg’s motivation-hygiene theory which stated that “the more employees are motivated by supervisor support and other factors, the more they prevent dissatisfaction” [30]. Therefore, if employees were satisfied with their supervisor’s support of their work, they would be accessible and punctual for their job in whatever type of health institution they are being employed. It was also in line with the theory of Adam’s equity which stated that “if there is equity in the outcome to input ratio of an employee to other employees, the more likely to be satisfied” [28].

Moreover, the findings of this study stated that health professionals who had good managers’ attitudes towards time were more likely to have good ATM than their counterparts. This finding was supported by Pickle Jar’s time management theory which stated that “having a good attitude to manage distractions is the best way to manage our time, which in turn leads to goal achievement” [31].

### Strengths and limitations of the study

Despite having typical limitations, this study tried to address extensively ATM by incorporating important variables which have been missed in previous studies such as work autonomy, workload, managers’ attitude towards time, profession, living conditions, co-worker relationships, professional development, and working unit Besides, we didn’t separately study ATM across specific health professionals category which might affect a clearer picture of the relationship between ATM and profession.

The use of self-administered questionnaires may have some potential for reporting biases. This study cannot determine the cause-and-effect relationship due to the cross-sectional nature of the study design. It would be much more informative and representative if it had been conducted in a multi-sectoral setting. Statistical reports are limited in the introduction and other relevant sections of this study due to a lack of research articles concerning this title. Associated factors of ATM have been identified in this study. But it would be more strong evidence if those factors were explored further using a qualitative study. It would be much more impressive if time management practice and its associated factors had been studied besides attitude towards time management.

## Conclusions

The attitude towards time management in public hospitals of Dessie City was low. Satisfaction with organizational policies and strategies, satisfaction with supervisor support, and managers’ good attitude towards time management were significantly associated factors in public hospitals.

Policymakers need to emphasize their policies and strategies in a way to break the bottleneck of dissatisfaction of employees, focusing on supervisor support and organizational policy and strategy. Hospital managers are expected to have a good attitude towards time to be role models for employees which helps them to develop a good attitude towards time. Health professionals are expected to deal with their directors about the factors which enforce them to be dissatisfied with the job. Further study concerning time management practices is needed to address the problem extensively through incorporating observational and follow-up studies supported by a qualitative method in different settings.

## Data Availability

The results of this study were analyzed using the collected primary data. All relevant data are included in this paper.

## Abbreviations

ATM: Attitude Towards Time Management
IRB: Institutional Review Board
SPSS: Statistical Package for Social Sciences
TMP: Time Management Practice
UoG: University of Gondar
WHO: World Health Organization
